# CASA: a comprehensive database resource for the COVID-19 Alternative Splicing Atlas

**DOI:** 10.1186/s12967-022-03699-8

**Published:** 2022-10-20

**Authors:** Yaxin Chen, Gang Wang, Jingyi Li, Lei Xia, Lin Zhu, Wenxing Li, Qiang Luo, Yinlu Liao, Yao Lin, Liyun Bi, Hubin Chen, Jiemei Chu, Yueqi Li, Jinming Su, Li Ye, Jun-jun Jiang, Hao Liang, Weimin Li, Sanqi An

**Affiliations:** 1 Frontiers Science Center for Disease-Related Molecular Network, Precision Medicine Research Center, West China Hospital, Department of Respiratory and Critical Care Medicine, Sichuan University, Chengdu, Sichuan China; 2 Biosafety Level-3 Laboratory, Life Sciences Institute & Guangxi Collaborative Innovation Center for Biomedicine, Guangxi Medical University, Nanning, 530021 Guangxi China; 3 Institute of Hydrobiology, Chinese Academy of Sciences, Wuhan, Hubei China; 4 Department of Biochemistry and Molecular Biology, School of Basic Medical Sciences, Southern Medical University, Guangzhou, 510515 Guangdong China

## Abstract

**Background:**

As a key process in transcriptional regulatory mechanisms, alternative splicing (AS) plays a crucial role in maintaining the diversity of RNA and protein expression, and mediates the immune response in infectious diseases, especially for the COVID-19. Therefore, urgent data gathering and more research of AS profiles in microbe-infected human cells are needed to improve understanding of COVID-19 and related infectious diseases. Herein, we have created CASA, the COVID-19 Alternative Splicing Atlas to provide a convenient computing platform for studies of AS in COVID-19 and COVID-19-related infectious diseases.

**Methods:**

In CASA, we reanalyzed thousands of RNA-seq datasets generated from 65 different tissues, organoids and cell lines to systematically obtain quantitative data on AS events under different conditions. A total of 262,994 AS events from various infectious diseases with differing severity were detected and visualized in this database. In order to explore the potential function of dynamics AS events, we performed analysis of functional annotations and drug-target interactions affected by AS in each dataset. RNA-binding proteins (RBPs), which may regulate these dynamic AS events are also provided for users in this database.

**Results:**

CASA displays microbe-induced alterations of the host cell splicing landscape across different virus families and helps users identify condition-specific splicing patterns, as well as their potential regulators. CASA may greatly facilitate the exploration of AS profiles and novel mechanisms of host cell splicing by viral manipulation. CASA is freely available at http://www.splicedb.net/casa/.

**Supplementary Information:**

The online version contains supplementary material available at 10.1186/s12967-022-03699-8.

## Background

Long COVID-19 has now been shown to have a lasting impact on people’s lives [1, 2]. As new variants of COVID-19 and other viruses emerge, COVID-19 vaccine breakthroughs urgently require researchers to fully explore the pathogenic mechanisms of COVID-19 infection [3]. At present, alternative splicing (AS), as one of the most important mechanisms to regulate gene expression and produce proteome diversity in the host, has attracted increasing attention in COVID-19 studies [4–8].

Previous studies have suggested that the immune response in COVID-19 patients is similar to that of many infectious diseases, including SARS-CoV, human immunodeficiency virus (HIV), influenza A virus (IAV), human cytomegalovirus (HCMV), herpes simplex virus (HSV), dengue virus 2, bacterial infections and viremia [9–12]. Recent studies have demonstrated that hundreds of host genes show alternative mRNA splicing in COVID-19 and other infectious diseases [4, 11, 13, 14]. Being relevant to host gene regulation, AS is a key aspect of virus-host interaction and plays an essential role in the host immune response to microbial infection [15]. For instance, the accessibility of hnRNP K transcripts is altered by IAV, triggering alterations in host cell splicing that promote IAV replication [16]. RNA splicing in host cells is also inhibited by the Vpr protein of HIV-1 and the NS5 protein of dengue virus [17]. Guttman et al. found that global mRNA splicing was altered due to the interaction between SARS-CoV-2 proteins and host mRNA, which consequently suppressed host defenses [4]. It was also reported that the extent of splicing deregulation in the host was associated with the severity of COVID-19 and affected drug-protein interactions [7]. In addition, it is widely accepted that viral infection-induced AS of host RNAs can alter the translation of proteins in the host cell [7, 12].

There are several databases focused on AS in various cancers and during cell development in different species [18, 19]. A database focused on interpreting the profiles of RNA expression, proteins, metabolites and lipid levels in COVID-19 promotes research on AS mechanisms related to COVID-19 [20]. However, even though AS plays a very essential role in COVID-19 infection as reported, there is a lack of resources focusing on AS profiles under different conditions of COVID-19 infection which could otherwise facilitate AS-related research in COVID-19.

To address this issue, we present CASA, a comprehensive database featuring the COVID-19 Alternative Splicing Atlas. In CASA, thousands of samples with RNA-seq data and related clinical/phenotype information were manually collected, integrated and reanalyzed. CASA comprises data on AS events for 12 microbes (e.g., Alpha, Delta, and Omicron variants of SARS-CoV-2, SARS-CoV, HIV, influenza A virus, HCMV, HSV-1, dengue virus 2, bacteria, and viremia), each sampled from different tissues, organoids or cell lines, and presents information regarding conditions affecting AS such as disease severity (e.g., mild, moderate or severe COVID-19), cell types and infection time [21]. To explore the potential function of microbe-induced AS, a series of tools are embedded in CASA, such as functional annotation for AS, drug-target interactions, identification of RNA-binding proteins (RBPs) and the distribution of AS on chromosomes. CASA is structured around condition-specific aspects of AS and is freely available at http://www.splicedb.net/casa/ with an interface that allows users to search by microbe name, tissue, region and feature (e.g., project ID, symbol, AS type). The database provides details on AS events of interest and can be visualized at the inclusion level under specific conditions.

## Materials and methods

### Data collection

RNA sequencing data were obtained from the Sequence Read Archive (SRA, https://www.ncbi.nlm.nih.gov/sra) of the National Center for Biotechnology Information. We selected the following keywords for the search: (‘COVID-19’[Selection]) OR (‘SARS-CoV-2’[Selection]) AND (‘Human’[Organism]) AND (‘Bioproject sra’[Filter]) AND (‘RNA-seq’[Strategy]). Through the search, we initially selected 102 BioProjects for further analysis. To filter out the low-quality data, only the data with mapping rate > 70% and data sequencing depth > 50 M reads per sample were considered for the further analyses. Finally, there are 59 BioProjects with a total of 2,163 samples without prior permission for construction of the database can be free download. The types of viruses covered by the data include Alpha, Delta, and Omicron variants of SARS-CoV-2, seasonal coronavirus, influenza virus, HCMV, dengue virus, HSV and HIV, among which the most representative is SARS-CoV-2. Meanwhile, all the data were from humans and generated by the Illumina platform.

### Differential AS analysis

The reads generated by RNA-seq were mapped to the hg38 human reference genome using HISTA2 with default values for parameters (version 2.1.0) [22]. The SAM format was converted to BAM and sorted by SAMtools with default values for parameters (version 1.1) [23]. We grouped the data separately by disease type, stage, treatment, time, age and other influencing factors, and used the sorted bam as input data to analyze AS by using the rMATs algorithm [24]. The parameters were: “nthread” = 8, “cstat” = 0.0001. Five classical AS types including skipped exon (SE), alternative 5′ splice site (A5SS), alternative 3′ splice site (A3SS), mutually exclusive exons (MXE) and retained intron (RI) were included in the database as we descripted before [5]. Differential AS events between two groups were identified as the following criterion: (i) number of reads > 5; (ii) FDR ≤ 0.15 according to our previous research [5]. We selected the JCEC (Junction Counts and Reads on target Exon Counts) file, which includes the additional exon count data as the result files, where lncLevels1 and lncLevels2 correspond to the exon inclusion levels calculated from normalized counts of each biological repeat in two different treatments. lncLevelDifference represents the difference between lncLevels1 and lncLevels2; if the difference is large and significant, it presumed to be the result of AS. To make mechanism more consistent between parameters in CASA and newly submitted datasets, we added detailed description documents in http://www.splicedb.net/casa/gseRun/document. Users can select file type, read lengths, sequencing type and library type according their own data situation, these parameters will not affect the mechanism for consistency between our parameters and newly submitted datasets.

### GO function enrichment and KEGG pathway analysis

To further investigate the relevant mechanisms that influence AS in COVID-19 patients, based on the false discovery rate (FDR) ≤ 0.1, we used the differential AS genes to perform gene ontology (GO) functional enrichment analysis and Kyoto Encyclopedia of Genes and Genomes (KEGG) pathway enrichment analysis in DAVID (https://david.ncifcrf.gov/) [25] and took the top 15 items ranked in FDR ascending order as the results. The GO enrichment analysis included cellular component, molecular function and biological process [26].

### RBP map analysis

One of the important posttranscriptional regulatory roles of RBPs is the regulation of AS. The web server, RNA Map Analysis and Plotting Server (rMAPS2, http://rmaps.cecsresearch.org), can identify candidate RBPs that control regulated AS events in specific biological processes and reveal their potential position-dependent effects on exon splicing [27, 28]. We used rMAPS2 to analyze the binding sites around differential AS events for over 100 known RBPs. The five AS events (SE, A5SS, A3SS, MXE and RI) previously calculated were used as input files. The options rMAPS used were as follows: Genome Assembly = hg38, Plot Type = Motif Score and p-Val Combined, Intron = 250, Exon = 50, Sliding Window Size = 50 and Interval = 1.

### Database implementation

CASA was organized using MySQL (version 10.1.48), and the web interface was developed based on the MVC (Model View Controller) architecture pattern. ElementUI (version 2.15.7) was adopted to design web pages. Apache Groovy programming was used for data processing and application operations. Echart (version 5.3.0), Vue (version 2.6.14) and D3 (version 7.4.4) were employed for data visualization. The CASA database has a convenient web interface to search, browse and download data about COVID-19-related AS events. The platform is publicly accessible through the website http://rnaspace.net/casa/.

## Results

### Usage and examples

#### Data content and access

**Fig. 1:**
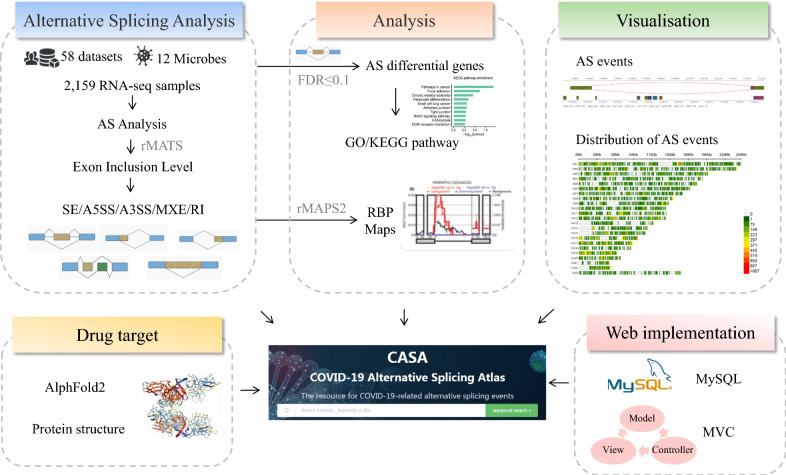
Overview of database construction. Alternative splicing event quantification and identification in CASA were processed with all RNA-seq libraries using standard pipelines

CASA is a database to present collections and annotations of microbe-induced AS in humans. Currently, CASA includes a total of 2163 human samples from 59 independent datasets, covering 15 body sites, 12 microbes and 15 countries/regions (Fig. 1). For these samples, most (64.47%, 1392/2159) were generated from tissue, and the second most frequent source of samples (28.35%, 612/2159) were cell lines. The most abundant body sites in the atmosphere were blood, lung and colon, and the top three countries providing samples were the USA (56.14%, 1212/2159), France (19.92%, 430/2159) and Spain (4.72%, 102/2159) (Additional file 1: Table S1). Samples from tissues, organoids or cell lines were collected under different conditions involving disease severity (mild, moderate or severe COVID-19), infection time, therapy and variant information, including the presence of Alpha, Delta, and Omicron variants. This related phenotype/clinical information helps us infer the condition-specific splicing patterns, as well as their potential regulators.

In total, five classical splicing types were considered, and 7,656,845 AS events detected from bulk RNA sequencing data are available in CASA, where the most frequently detected events were SE and MXE, accounting for more than 80% of the AS events (SE: 69.75%; MXE: 12.45%; Additional file 2: Table S2). The distribution of different types of AS events in each body site is summarized in Additional file 3: Fig. S1. For samples with group information, significant differential splicing patterns were identified, resulting in 262,994 differential events. Among these events, 1.51% (3,935/262,994) were sample-specific. Notably, 11 genes with differential splicing patterns induced by viral infections were manually curated from the published literature and were also deposited in CASA (Additional file 2: Table S2), suggesting CASA is a reliable database for AS-related research.

#### Database interface

**Fig. 2:**
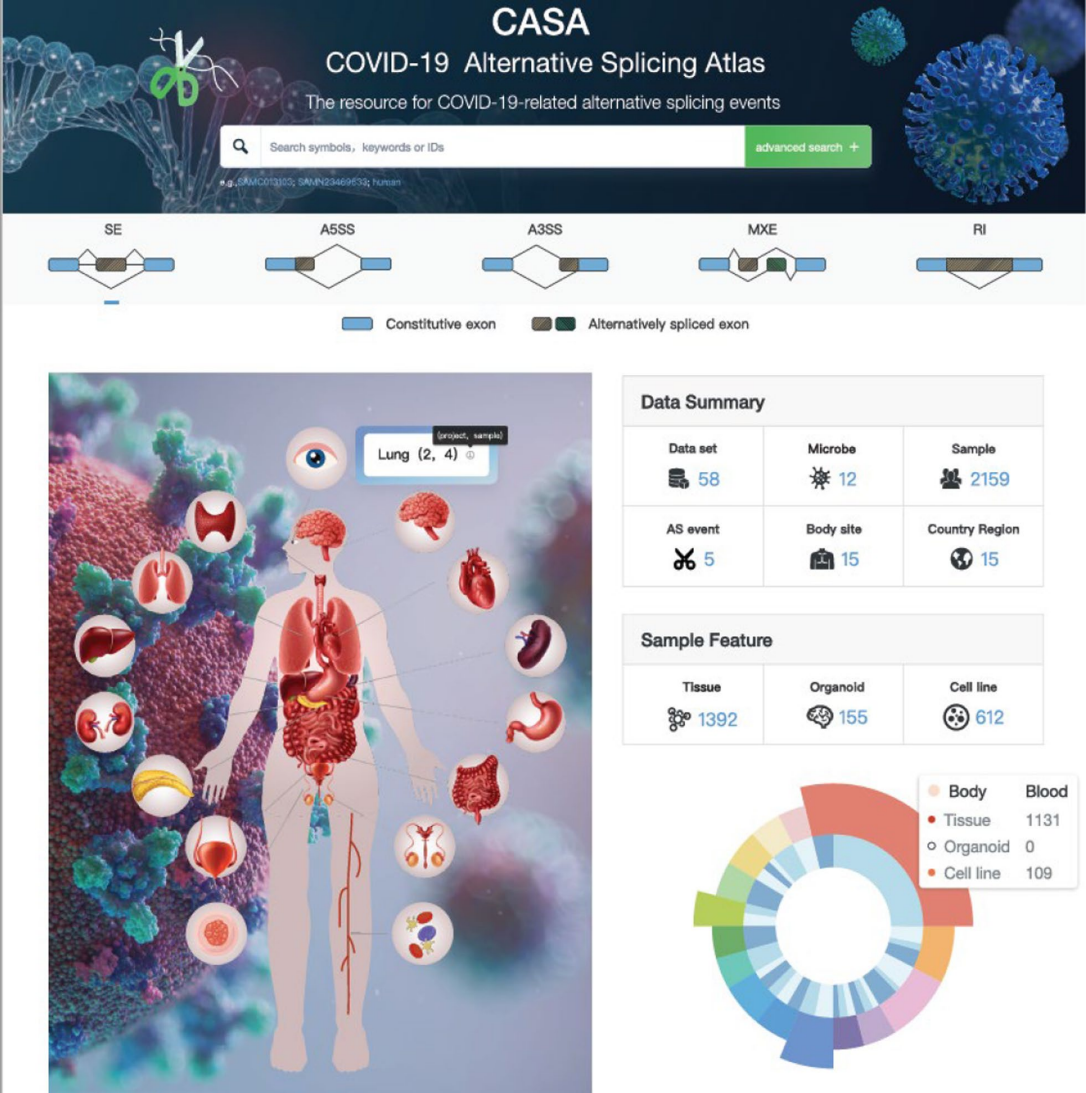
The data landscape and web interface of CASA

There are four major modules provided in CASA: (i) ‘Search’, a retrieval model for querying the AS events under specific conditions; (ii) ‘Browse’, a module that shows the statistical results from all the datasets in CASA; (iii) ‘Toolkit’, a module containing practical functions, including the analysis of differentially regulated alternative exons under different conditions; and (iv) ‘Document’, detailed introduction and tutorials for CASA. The data landscape of CASA is shown in Fig. 2, including the distribution of datasets across body sites and the number of samples from tissues, organoids and cell lines in humans.

#### Database queries

**Fig. 3:**
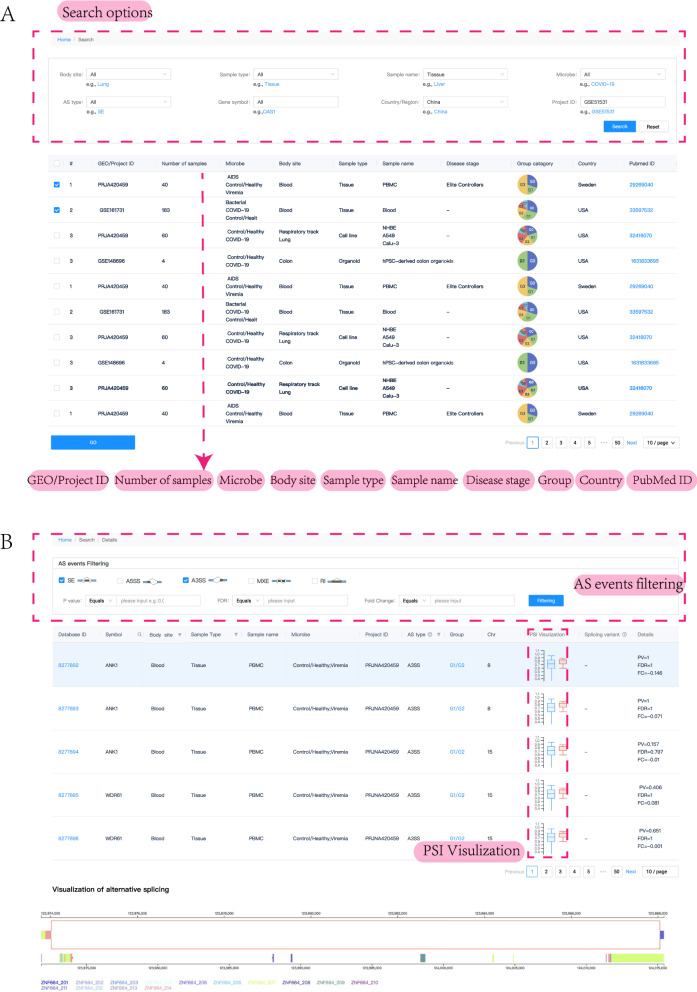
CASA search interface. **A** Users can search for alternatively spliced exons by body site, sample type, sample name, microbe, project ID and gene symbol. **B** Details of AS events from one or more datasets. Users can click on the “Database ID” links to the basic information of the AS event

The search interface of CASA is shown in Fig. 3A. User-friendly web interfaces are provided in CASA, where users can retrieve splicing events across all datasets by freely combining different search criteria, such as specific sample type, sample name, microbe, gene symbol or dataset. The multi-condition advanced search and keyword search on the home page are also available (Fig. 2). Furthermore, a series of smart auxiliary widgets on the details page are offered for downstream analysis, such as a query search bar and sorting functionality.

To maximize utility and allow user-specified thresholds in CASA, we present all splicing results in an unfiltered form. Consequently, we recommend that users screen records for quality and filter them on the basis of exon inclusion ratio, FDR and a minimum number of inclusion and exclusion reads per exon. All search results are available in tab-separated text. We also recommend that users cross-validate results from CASA. More details on how to search the database and how to browse the details of the search results can be found in the ‘Help’ part of the ‘Document’ page.

**Fig. 4:**
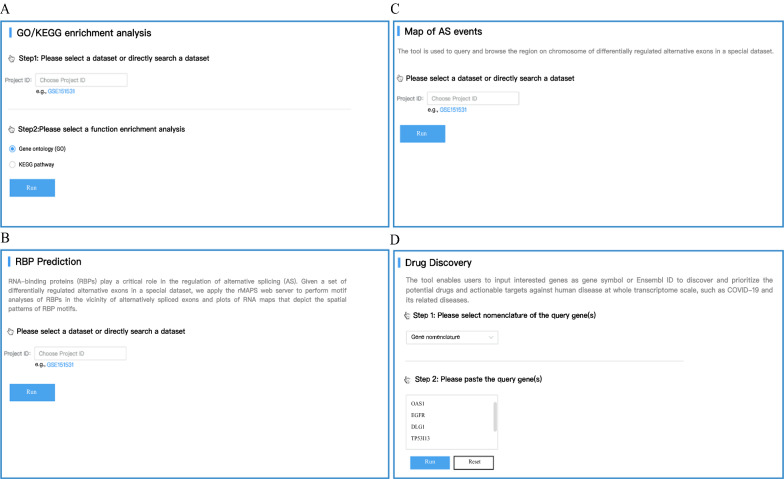
Toolkits in CASA

To explore the potential function of observed changes in AS events, a series of analysis results were integrated into CASA, such as motif analysis of RBPs in the vicinity of alternatively spliced exons, which was provided to explore whether an RBP could act as a potential regulator for AS events occurring under special conditions (Fig. 4A). In addition, the results of GO/KEGG pathway enrichment analysis and the distribution of AS events on chromosomes were also integrated into the database (Fig. 4B, C). For genes with differential expression or for differentially regulated alternative exons, we provide two resources to identify potential drugs and actionable targets for genes of interest: drug-target-pathway interactions based on the KEGG pathways, and also DrugCentral, which was embedded in CASA. In addition, a drug discovery tool was developed as well (Fig. 4D).

#### AS analysis of researcher own transcriptomic data

In order to better support researchers’ exploration of AS in disease, CASA also is designed to help researchers perform AS analysis of their own RNA-seq data. Researchers only need to upload their RNA-seq data and set values for parameters according their own data situation, the tool will systematically analyze AS automatically, and all these results can then be downloaded. After the analysis is completed, a new web-page will pop up to allow investigators to conduct 14 different analyses on their uploaded data privately. It also contains the functional enrichment information with GO and KEGG pathway analysis. Furthermore, these results can be downloaded and directly saved as a PDF file with high resolution. A detailed tutorial is available for the database at http://www.splicedb.net/casa/gseRun/document.

#### Case study: using CASA to discover AS of OAS1 is associated with COVID-19 risk

**Fig. 5:**
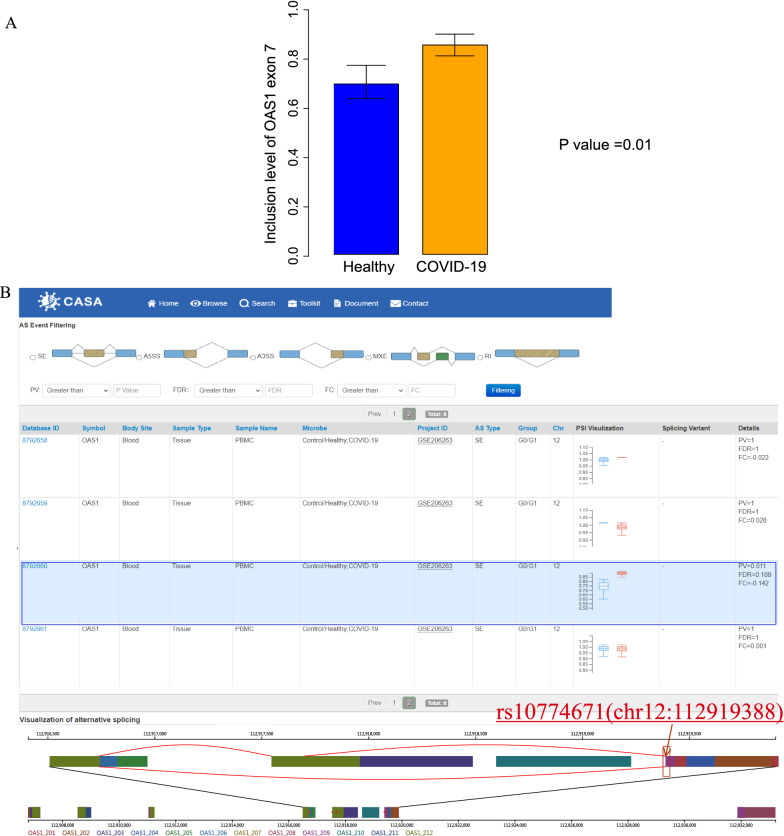
An example of the inclusion level of an exon within the COVID-19 infection-associated gene OAS1. **A** COVID-19 patients in different groups show significantly different PSI levels of OAS1. **B** Red box shows different PSI level of OAS1. The blue box shows the significantly alternative splicing regions contain SNP site rs10774671 (red arrow pointing down), which was previously reported to be associated with COVID-19 infection and alternative splicing events, indicating CASA is a reliable database

Splicing patterns of several hundred human genes are associated with viral/bacterial infection [13]. For example, the splicing pattern of OAS1 was previously reported to be associated with severe COVID-19 infection [8]. OAS genes encode enzymes which activate ribonuclease L and play essential roles in several antiviral mechanisms. A variant rs10774671 (chr12:112919388) falls in a splice acceptor site at exon 7 of OAS1 and changes the activation of the OAS1 enzyme [8]. To be more specific, the A allele of rs10774671 result in the AS of exon 7 of OAS1 pre-mRNA to produce different protein variants, causing virus infectivity [29]. Exon 7 inclusion levels of OAS1 between healthy and COVID-19 are shown in Fig. 5A, we found the Exon 7 inclusion levels of OAS1 was significantly changed between healthy and COVID-19 (FDR = 0.1). Using CASA, we also found the exon 7 of the OAS1 gene shows a different inclusion level at chr12:112919388 between healthy and COVID-19, the reference SNP cluster ID rs10774671 was included in alternative splicing event (Fig. 5B). Therefore, this alternative splicing event would change the SNP site rs10774671.This result from CASA is consistent with previous findings that OAS1 splicing pattern was associated with COVID-19 susceptibility and severity [8, 30–34], indicating CASA is a reliable and accurate database for AS-related research.

## Discussion and conclusions

A broad program of AS of host genes tends to be induced when human cells are infected with microbes, including SARS-COV-2, SARS-CoV, HIV, influenza A virus, HCMV, HSV-1, and dengue virus 2 [9]. There is an urgent need to investigate the role of AS in the mechanism of these infectious diseases. Several cancer- and development-related AS databases have been developed and are widely used by researchers [12, 13, 23, 24], contributing to our knowledge of AS mechanisms in cancer and developmental biology. Herein, we developed CASA, the first comprehensive AS database for COVID-19 and COVID-19-related infectious diseases, to provide comprehensive AS information regarding different tissues in COVID-19 and nine other infectious diseases. In addition, potential RBP-regulated AS as well as corresponding functions enriched in AS are also displayed.

In the future, we promise that CASA will be kept up to date as follows: (i) Additional online tools will be provided in CASA based on user feedback, and (ii) we promise to integrate more samples and more infectious diseases into CASA and will continue to update the database every six months. New datasets released after May 2022 will be integrated and reanalyzed to ensure their value as user-friendly COVID-19-related AS databases. We believe that CASA will motivate research on the functions of virus-induced splicing alterations in host cells in terms of cell biology, as well as mechanisms involving AS in infectious diseases.

## Supplementary Information

**Additional file 1: Table S1**. Summary of samples in CASA.

**Additional file 2: Table S2**. Summary of alternative splicing records in CASA.

**Additional file 3: Figure S1.** The landscape of samples and AS events in CASA. (A) The number of samples in each body site. (B) Proportion of different splicing types in CASA. (C-D) The number and distribution of different splicing types across body sites.

## Data Availability

All the data presented in this study are available on CASA (http://www.splicedb.net/).
