# Supplementary figures and images for: CASA: a comprehensive database resource for the COVID-19 Alternative Splicing Atlas
Source: J Transl Med. 2022 Oct 20;20:473. doi: 10.1186/s12967-022-03699-8 (PMC9583055; doi:10.1186/s12967-022-03699-8)

A

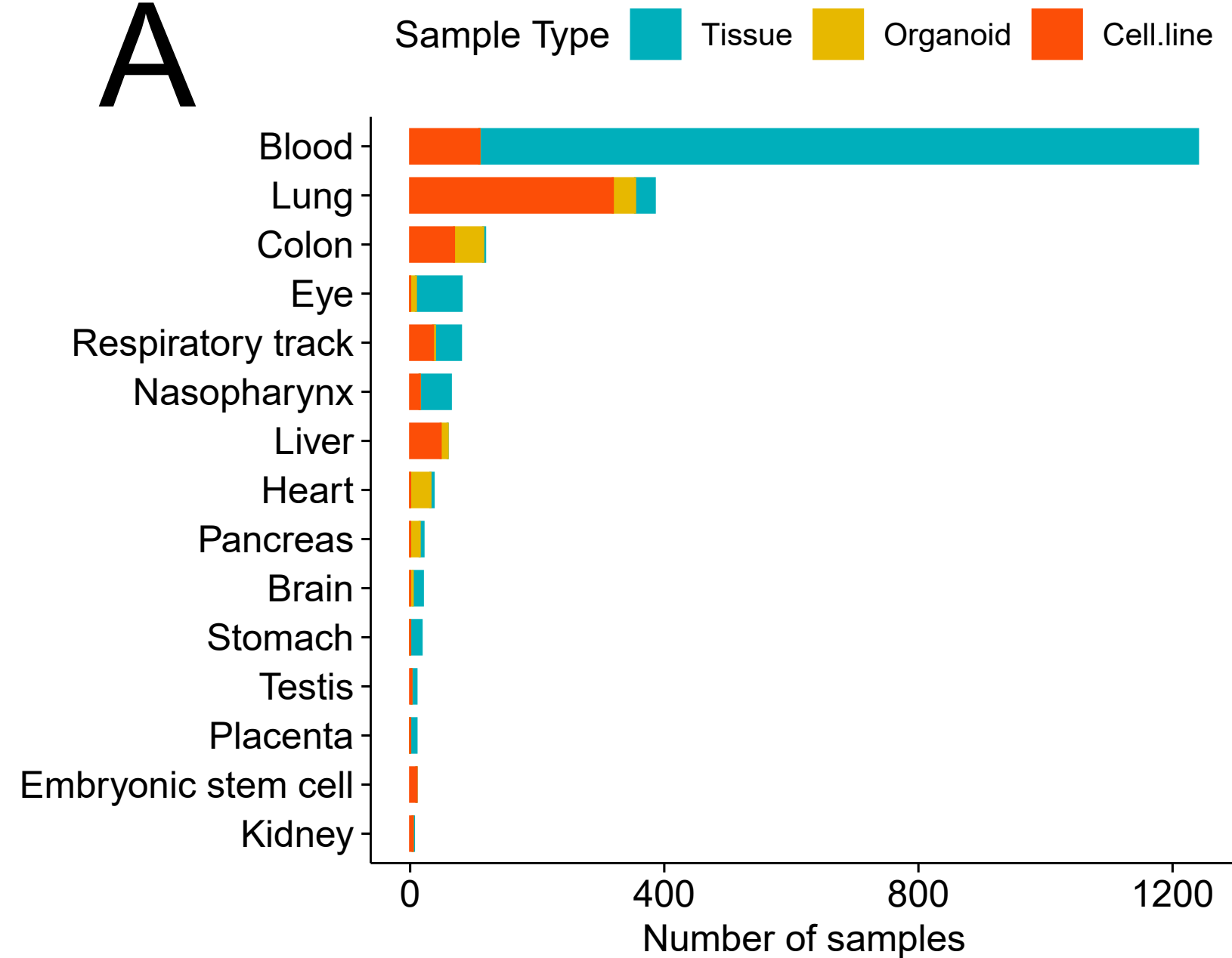

B

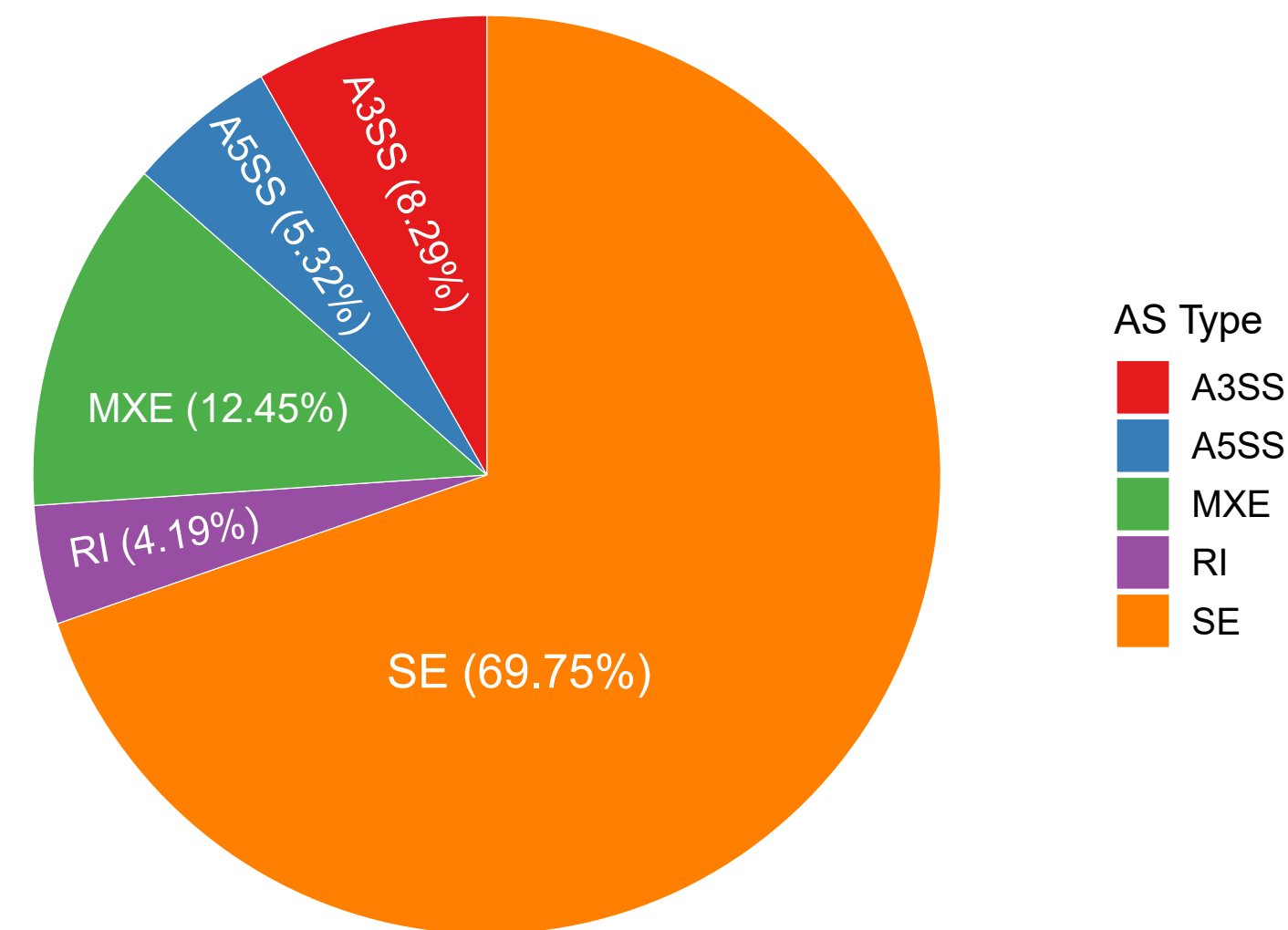

C

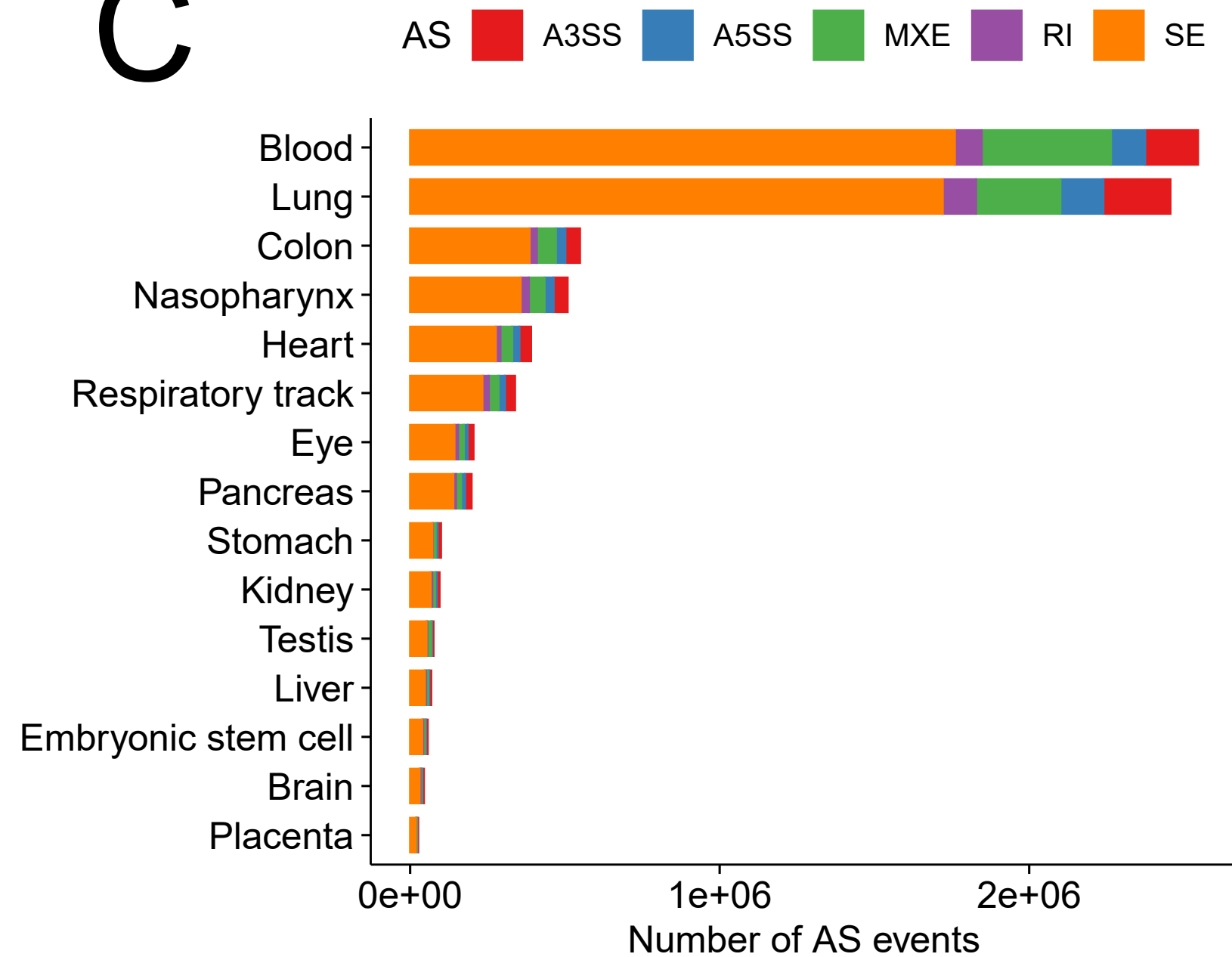

D

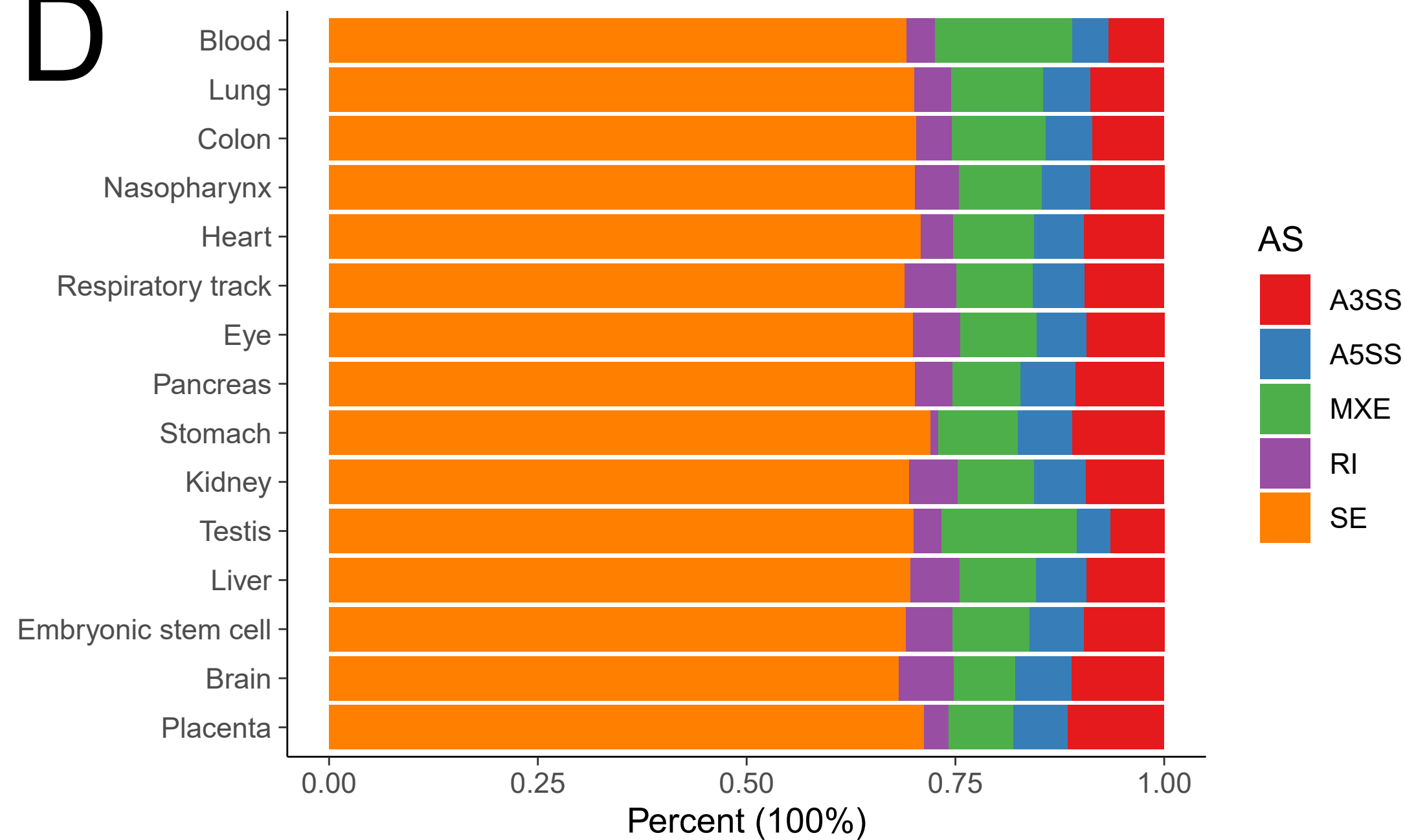

Supplement: Supplementary file 3 — Additional file 3: Figure S1. The landscape of samples and AS events in CASA. (A) The number of samples in each body site. (B) Proportion of different splicing types in CASA. (C-D) The number and distribution of different splicing types across body sites. [file 12967_2022_3699_MOESM3_ESM.pdf]
